# Impact of antiretroviral therapy in primary HIV infection on natural killer cell function and the association with viral rebound and HIV DNA following treatment interruption

**DOI:** 10.3389/fimmu.2022.878743

**Published:** 2022-08-30

**Authors:** Matthew Pace, Ane Ogbe, Jacob Hurst, Nicola Robinson, Jodi Meyerowitz, Natalia Olejniczak, John P. Thornhill, Mathew Jones, Anele Waters, Julianne Lwanga, Kristen Kuldanek, Rebecca Hall, Panagiota Zacharopoulou, Genevieve E. Martin, Helen Brown, Nneka Nwokolo, Dimitra Peppa, Julie Fox, Sarah Fidler, John Frater

**Affiliations:** ^1^ Nuffield Department of Medicine, University of Oxford, Oxford, United Kingdom; ^2^ Etcembly Ltd, Harwell Campus, Didcot, United Kingdom; ^3^ Blizard Institute, Barts and The London School of Medicine and Dentistry, Queen Mary University of London, London, United Kingdom; ^4^ Department of Infection, Guys and St Thomas’ National Health Service (NHS) Trust, London, United Kingdom; ^5^ Department of HIV Medicine, St Mary’s Hospital, Imperial College Healthcare National Health Service (NHS) Trust, London, United Kingdom; ^6^ Department of Infectious Diseases, Monash University, Melbourne, VIC, Australia; ^7^ Department of HIV/GUM, Chelsea and Westminster Hospital, London, United Kingdom; ^8^ Division of Infection and Immunity, University College, London, United Kingdom; ^9^ Department of Infectious Disease, Faculty of Medicine, Imperial College London, London, United Kingdom; ^10^ National Institute for Health and Care Research (NIHR) Imperial College Biomedical Research Centre, London, United Kingdom; ^11^ National Institute for Health and Care Research (NIHR) Oxford Biomedical Research Centre, Oxford, United Kingdom

**Keywords:** HIV-human immunodeficiency virus, NK cell, viral rebound, antiretroviral (ARV), treatment interruption (TI)

## Abstract

Natural Killer (NK) cells play a key role in controlling HIV replication, with potential downstream impact on the size of the HIV reservoir and likelihood of viral rebound after antiretroviral therapy (ART) cessation. It is therefore important to understand how primary HIV infection (PHI) disrupts NK cell function, and how these functions are restored by early ART. We examined the impact of commencing ART during PHI on phenotypic and functional NK cell markers at treatment initiation (baseline), 3 months, 1 year, and 2 years in seven well-characterised participants in comparison to HIV seronegative volunteers. We then examined how those NK cell properties differentially impacted by ART related to time to viral rebound and HIV DNA levels in 44 individuals from the SPARTAC trial who stopped ART after 48 weeks treatment, started during PHI. NK cell markers that were significantly different between the seven people with HIV (PWH) treated for 2 years and HIV uninfected individuals included NKG2C levels in CD56^dim^ NK cells, Tim-3 expression in CD56^bright^ NK cells, IFN-γ expressed by CD56^dim^ NK cells after IL-12/IL-18 stimulation and the fraction of Eomes-/T-bet+ in CD56^dim^ and CD56^bright^ NK cells. When exploring time to viral rebound after stopping ART among the 44 SPARTAC participants, no single NK phenotypic marker correlated with control. Higher levels of IL-12/IL-18 mediated NK cell degranulation at baseline were associated with longer times to viral rebound after treatment interruption (P=0.028). Additionally, we found higher fractions of CD56^dim^ NK cells in individuals with lower levels of HIV DNA (P=0.048). NKG2A and NKp30 levels in CD56^neg^ NK cells were higher in patients with lower HIV DNA levels (p=0.00174, r=-0.49 and p=0.03, r= -0.327, respectively) while CD27 levels were higher in those with higher levels of HIV DNA (p=0.026). These data show NK cell functions are heterogeneously impacted by HIV infection with a mixed picture of resolution on ART, and that while NK cells may affect HIV DNA levels and time to viral rebound, no single NK cell marker defined delayed viral rebound.

## Introduction

Despite the effectiveness of antiretroviral therapy (ART) in controlling and preventing HIV infection, efforts are still focusing on a cure to overcome the stigma and the disadvantages of lifelong therapy (1). Recent cure trials suggest that while a sterilising cure will be extremely difficult, a period of ART-free viral control may be feasible (2). In a small subset of individuals termed ‘post treatment controllers’ (PTCs), stopping ART is not followed by immediate viral rebound in plasma (3). Understanding the immunological mechanisms that underlie a PTC phenotype may provide important targets for cure efforts.

Elite controllers (EC), who spontaneously control HIV without therapy for more than 6 months, frequently have well-defined, strong anti-HIV CD8 T cell responses and are enriched for protective HLA Class I alleles (4) (5). In addition, animal studies exploring PTC following broadly neutralising antibody (bNAb) therapy have identified CD8 T cells as potential mediators of long-lasting viral control (6, 7). Natural killer (NK) cells may also be a potential effector of PTC. Similar to CD8 T cells, NK cells have the ability to clear infected cells through multiple mechanisms including exocytosis of cytotoxic granules leading to lysis, signalling through TNF death receptors and antibody dependent cell cytotoxicity (8, 9). The application of this function to HIV cure has gained traction as animal studies using bNAbs against HIV (10) have suggested a role for NK cells to augment responses targeting the HIV reservoir (11). Animal models of non-pathogenic SIV corroborate this NK cell potential as they appeared to control SIV replication in the lymph nodes of African green monkeys (12). In humans, properties of NK cells including NK cell subset distribution, activation and the expression of activating NK receptors have correlated with changes in HIV DNA reservoir size and decline (13, 14).

A role for NK cells in PTC has also been inferred from several studies indicating that PTCs are immunologically distinct from elite controllers (EC), and therefore may not rely on CD8 mediated control (15). Unlike ECs, PTCs do not have strong CD8 specific responses (3) or an enrichment of protective CD8 HLA Class I alleles (3, 16). The exact mechanisms of PTC - or clinical markers identifying these individuals - remain undetermined. Understanding the factors that contribute to viral rebound and lower HIV DNA levels could therefore help determine factors important for post-ART control.

As HIV infection is likely to disrupt NK cell functionality, any restorative effect of ART may provide important insights into specific cell phenotypes and properties that need to be sustained for viral control. Several studies have examined the effect of ART on NK cell function and phenotype with discrepancies regarding the ability of therapy to restore NK cell recovery after HIV infection (reviewed in (8, 17)). While many studies suggest NK cells recover after ART, some have suggested that not all NK cell parameters return to their pre-HIV state (17). However, the majority of these studies were performed in chronically treated HIV individuals (18, 19), after several years of ART, or without longitudinal data (20).

To address these issues in primary HIV infection - where PTC is more frequently reported - (3, 21), we undertook a study of PWH who commenced treatment shortly after seroconversion in which some subsequently undertook a treatment interruption. The study was devised in two halves – first to examine whether early treatment could restore any NK dysfunction or phenotypic changes, and the second to evaluate if these NK-associated parameters were associated with time to viral rebound in participants who stopped ART. The ‘HIV Reservoir targeting with Early Antiretroviral Therapy’ (HEATHER) cohort comprised individuals starting and maintaining ART close to the time of HIV acquisition (22) and was key to the first half of the work, providing the opportunity to look in detail at longitudinal changes of NK cell properties after ART initiation in PHI. The Short-Pulse Anti-Retroviral Therapy at Seroconversion (SPARTAC) study (23) was the focus of the second half of the study and included a treatment interruption (with documented times to viral rebound and HIV DNA levels) allowing exploration of those NK cell phenotype and function parameters identified in the first part. By comparing the data from these two cohorts we aimed to determine whether NK cell markers that were impacted by early ART would also be those that mapped onto individuals showing evidence of PTC. If this was the case, it would provide a rationale for targeting NK cells in cure strategies.

## Materials and methods

### Participants

All participants gave written, informed consent. Recruitment for the HEATHER cohort was approved by the West Midlands—South Birmingham Research Ethics Committee (reference 14/WM/1104). HEATHER is a prospective observational cohort study of individuals who commence ART (and remain on uninterrupted therapy) within 3 months of the date of HIV diagnosis during PHI (22). Individuals are considered to have PHI if they meet any of the following criteria: HIV-1 positive antibody test within 6 months of a HIV-1 negative antibody test, HIV-1 antibody negative with positive PCR (or positive p24 Ag or viral load detectable), RITA (recent infection assay test algorithm) assay result consistent with recent infection, equivocal HIV-1 antibody test supported by a repeat test within 2 weeks showing a rising optical density or having clinical manifestations of symptomatic HIV seroconversion illness supported by antigen positivity. The time of seroconversion was estimated as the midpoint between the most recent negative or equivocal test and the first positive test for those who met relevant criteria, and as the date of test for all other participants. Individuals with co-existent active hepatitis B or C infection were not eligible for inclusion in HEATHER. CD4 count, CD8 count and VL were measured as part of routine clinical care with baseline VL and CD4 and CD8 counts taken as the earliest value prior to the initiation of ART. All blood samples were collected in (Acid Citrate Dextrose) ACD tubes.

SPARTAC (EudraCT Number: 2004-000446-20) was a multi-centre randomized controlled trial of short course antiretroviral therapy during PHI, which completed follow up in 2010. The full inclusion criteria and details of the SPARTAC trial are published elsewhere (23). PHI was defined and estimated date of seroconversion calculated similarly to the HEATHER cohort. In this work, we examined samples collected 12 weeks post ART initiation as that was the timepoint closest to baseline where samples were still available for analysis. For this study n = 44 participants who were randomly allocated to receive 48 weeks of ART are included. Ethical approval can be found in the Supplementary Methods section.

#### HIV-uninfected control populations

Eight healthy volunteers were recruited into the study as part of the HIV seronegative control group. PBMCs were isolated from these volunteers and cryopreserved for subsequent use in the NK cell phenotypic and functional panels. Demographic information for this cohort can be found in **Table 1**.

**Table 1:** Demographics of cohorts.

	HEATHER	SPARTAC	HIV-UNINFECTED
**Male**	7 (100%)	33 (75%)	3 (37.5%)
**Female**	0 (0%)	11 (25%)	5 (62.5%)
**Age (years)**	28 (27–33)*	34 (27.5-42.75)*	40 (34–51)*
**Ethnicity**	White: 4 (57.1%) Other: 3 (42.9%)	UK: 28 (63.6%) African: 11 (25%) Australian: 2 (4.5%) Other European: 3 (6.8%)	White: 5 (62.5%) Other: 3 (37.2%)
**Viral Subtype**	B: 3 (42.9%) CRF02-AG: 1 (14.3%) CRF02-AG/B: 1 (14.3%) CRF02-AG/G: 1 (14.3%) D/B: 1 (14.3%)	B: 33 (75%) C: 11 (25%)	
**Baseline CD4 (cells/μL)**	452 (331-554)*	643.5 (456-771.5)*	
**Baseline Viral Load (log(10) copies/mL)**	5.77 (4.73-6.73)*	4.66 (4.19-5.20)*	
**Time from seroconversion to randomisation (ART) (days)**	27 (21–33)*	90.5 (66-110.3)*	
**Time to Viral Rebound (400 copies/mL) (weeks)**	n/a	6 (4–40)*	
**Total HIV DNA (copies/million CD4 T cells)**	n/a	1749 (1087-2762)*	

* = interquartile range.

n/a, not applicable.

#### Cell culture

Frozen peripheral blood mononuclear cells (PBMC) were thawed and either immediately stained for phenotyping experiments or cultured for functional assays. For functional assays, PBMC were either cultured overnight in RPMI-1640 media with 10% FCS, L-glutamine and penicillin/streptomycin (R10) alone or were stimulated with 10ng/mL IL-12 (PeproTech, Rocky Hill USA) and 100ng/mL IL-18 (R&D Systems, Minneapolis USA). PBMC (both R10 and cytokine treated) were either co-cultured alone or with the K562 cell line at a 5:1 effector (PBMCs):target (E:T) ratio in a 96U bottom plate for 5 hours in the presence of CD107a PECy7 (Biolegend, San Diego USA). After 2 hours of co-culture GolgiStop and GolgiPlug (BD Biosciences, San Jose USA) were added to the cultures as per manufacturer’s recommendations. After co-culture, cells were washed and stained for flow cytometry.

#### Flow cytometry

For phenotyping experiments, cells were divided into an extracellular and intracellular panel. For the extracellular panel, cells were washed and stained at 4°C for 30 minutes with: Live/Dead fixable near-IR stain (ThermoFisher Scientific, Waltham USA), CD3 APC-Cy7 (HIT3a), CD14 APC-Cy7 (HCD14),CD19 APC-Cy7 (HIB19), CD56 BV421 (HCD56) [all Biolegend], CD16 FITC (CB16) [eBioscience, San Diego USA], NKG2A APC (REA110), NKG2C PEVio770 (REA205) [both Miltenyi Biotec, Bergisch Gladbach Germany], NKG2D PerCpCy5.5 (1D11), NKp30 PE (P30-15), NKp46 PE/Dazzle (9E2) [all Biolegend]. Cells were then washed twice with FACS buffer (PBS + 5% FCS + 1mM EDTA) and fixed with 2% formaldelhyde solution.

For the intracellular panel cells were washed and stained at 4°C for 30 minutes with: Live/Dead fixable near-IR stain (ThermoFisher Scientific), CD3 APC-Cy7 (HIT3a), CD14 APC-Cy7 (HCD14),CD19 APC-Cy7 (HIB19), CD56 BV421 (HCD56), CD16 PECy7 (3G8) [all Biolegend], Tim-3 PE (344823) [R&D Systems], CD27 Alexa Fluor 700 (O323), CD57 PE Dazzle (HNK-1), CD38 PerCpCy5.5 (HB-7) [all Biolegend]. Cells were then washed twice with FACS buffer. Cells were then fixed and permeabilised using the Human FoxP3 Buffer Set (BD Biosciences). Cells were first fixed in in 1x Buffer A for 10 minutes at room temperature. Cells were then washed with FACS buffer and resuspended in Buffer C for 30 minutes at room temperature. Cells were then washed twice with FACS buffer and then stained with EOMES eFluor 660 (WD1928) [eBioscience] and T-bet FITC (4B10) [Biolegend] at 4°C for 30 minutes.

For functional assays, cells were stained at 4°C for 30 minutes with: Live/Dead fixable near-IR stain (ThermoFisher), CD3 APC-Cy7 (HIT3a), CD14 APC-Cy7 (HCD14), CD19 APC-Cy7 (HIB19), CD56 BV421 (HCD56) [all Biolegend], and CD16 FITC (CB16) [eBioscience]. Cells were then washed twice with FACS buffer. Cells were permeabilised using BD FACS Permeabilizing solution 2 (BD Biosciences) for 10 minutes at room temperature. Cells were then washed once with FACS buffer and once with 1x Brilliant Stain Buffer (BD Biosciences). Cells were subsequently stained at 4°C for 30 minutes with: IFN-γ BV605 (BD Biosciences), TNF APC (Mab11), Granzyme A PerCpCy5.5 (CB9) [all Biolegend], Granzyme B Alexa Fluor 700 (GB11) [BD Biosciences], and Perforin PE (B-D48) [Biolegend] prior to being washed twice with FACS Buffer. All cells were run on a BD LSR II. Rainbow Calibration Particles (8 peaks) [BD Biosciences] were used to calibrate the machine to minimise daily variation.Total HIV DNA was measured as previously described (24). Briefly, CD4 T cells were enriched from frozen PBMC samples *via* negative selection (Dynabeads, Invitrogen, Carlsbad, CA). CD4 T cell DNA was then extracted (Qiagen, Venlo, Netherlands) and used as input DNA for PCR analysis.

#### CMV serology methods

Plasma from the SPARTAC cohort was taken from the 12 week timepoint and were tested for CMV IgG using the Abbott Architect i2000SR platform (Abbott, Chicago, IL, USA). The assay was performed by trained laboratory staff, in accordance with the manufacturer’s instructions, in a UK Accreditation Service (UKAS) accredited laboratory.

### Statistics

Analyses were performed and plots generated using GraphPad Prism (v7.0b). Except where otherwise specified, p-values < 0.05 were considered statistically significant. Simple comparisons were performed using parametric or non-parametric tests as appropriate and are described alongside the results and in figure legends. Datasets were explored both as continuous variables or as categorical variables stratifying data above/below median or mean values, accordingly. Longitudinal analysis was performed using GraphPad Prism version 9.1.2. A Kruskal-Wallis test with Dunn’s multiple comparisons test was performed comparing baseline values with 3 months post ART and uninfected controls and 2 years post ART with uninfected controls. Viral rebound after TI was defined as a plasma viral load >400 HIV RNA copies/ml on a least two consecutive measurements.

## Results

### Longitudinal effect of ART on NK cells following PHI

For the first component of the study, we aimed to identify those NK markers which were impacted by starting ART in PHI in the HEATHER cohort, with the aim - in the second part of the study - of seeing how these markers predicted time to rebound in a different PHI cohort (SPARTAC).

We identified seven participants from the HEATHER cohort who were all men who had sex with men (MSM), started ART during PHI and for whom longitudinal samples were available from before ART (baseline), 3 months, 1 year and 2 years post ART initiation. Their clinical characteristics are summarised in **Table 1**. The median age (interquartile range, IQR) was 28 (25–31). Their median CD4 T cell count was 452 cells/μL (286–607) and median viral load was 5.77 log copies/mL (4.73log-6.73log) at baseline. The median time from seroconversion to ART start was 27 days (21–23, 25–34).

**Figure 1:**
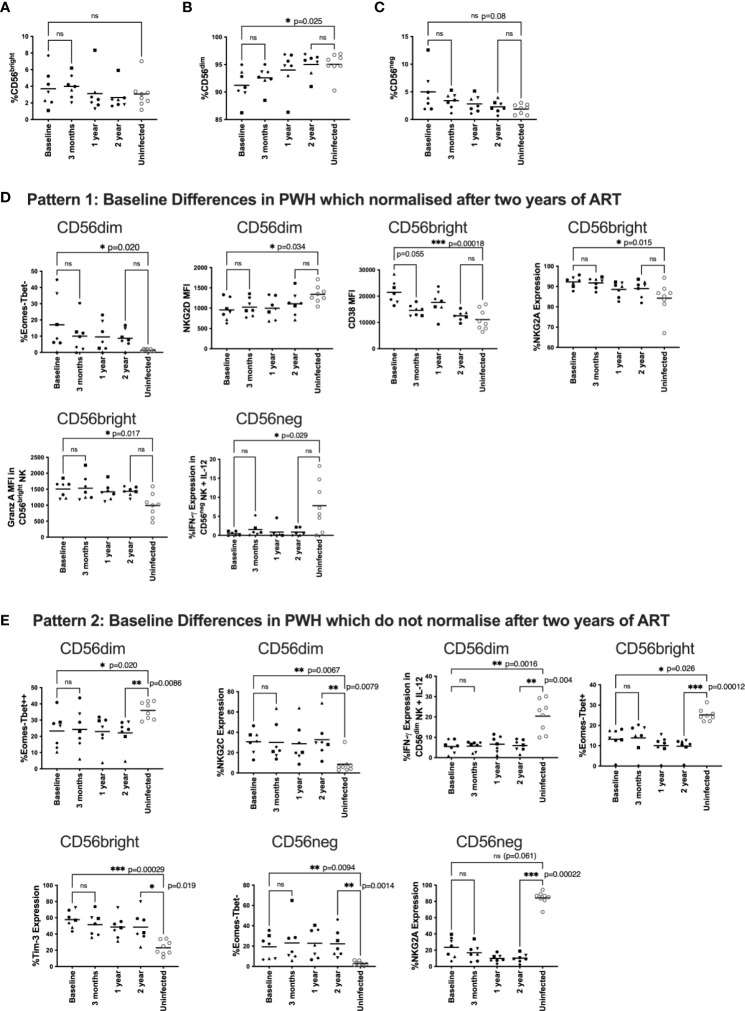
Changes to NK phenotype and function longitudinally on ART.PBMC from HEATHER samples were taken at baseline (off ART), 3 months, 1 year and 2 years post ART and compared to HIV uninfected individuals. HEATHER samples (n = 7) and HIV uninfected samples (n = 8) were stained for CD56 and analysed based on the expression of this marker into CD56^bright^, CD56^dim^, and CD56^neg^ NK cells at each timepoint **(A–C)**. Downstream analysis was performed on phenotypic and functional markers within the CD56 expressing populations. These are grouped to show those that were statistically different at baseline between PWH and HIV negative controls but then either corrected after two years of ART **(D)**, or remained statistically different after two years of ART **(E)**. For panels **(D, E)**, phenotypic and functional markers are presented as percent expression or median fluorescence intensity (MFI). For all data, only samples with statistical differences between HIV infected and HIV uninfected individuals are shown. A Kruskal-Wallis test with Dunn’s multiple comparison test was performed between baseline and 3 months ART, baseline and uninfected samples, and 2 year and uninfected samples. *P < 0.05; **P < 0.01; ***P < 0.001; ns not significant.

We first explored how NK cell function and phenotype were impacted by early HIV infection in comparison to HIV uninfected samples, and the extent to which this was reversed by ART over two years (**Figure 1**). Our HIV-uninfected participants had a median age of 40 (34–65) and were mostly female (5/8) (**Supplementary Table 1**).

First, we characterised NK cell subsets based on CD56 and CD16 expression including CD56^bright^, CD56^dim^, and CD56^neg^ NK cells. For clarity, for these and subsequent analyses in the HEATHER participants we focus on differences between baseline and two years (more detailed statistical comparisons for other timepoints are shown in Supplementary Material). The gating strategy is shown in **Supplemental Figure 1**. We found no statistical difference in the fraction of CD56^bright^ cells between PWH and HIV-uninfected individuals (**Figure 1A**) but there was a reduced fraction of CD56^dim^ NK cells in PWH at baseline that normalised post-ART initiation when compared to uninfected individuals (p=0.025, **Figure 1B**). Additionally, there was a trend towards higher CD56^neg^ NK cell frequencies in HIV-infected participants at baseline compared to uninfected individuals that was no longer apparent after ART (p=0.08, **Figure 1C**). We then examined the overall frequencies of NK cells per total lymphocytes and found no statistical difference over time (**Supplemental Figure 2**)

NK cells were first analysed by exploring their phenotypic markers and then by looking at cell function. We examined phenotypic markers including: the activation marker CD38; transcription factors T-bet and Eomes; the exhaustion marker Tim-3; phenotyping markers CD27 and CD57; the inhibitory receptor NKG2A; and the activating receptors NKG2C, NKG2D, NKp30, and NKp46. Gating strategies for these markers are shown in **Supplemental Figure 3**.

We tested NK cell function using two models: first by co-culturing cells with the MHC I negative cell line K562 as an NK target, and second by testing NK cell responsiveness to the cytokines IL-12 and IL-18. We stained NK cells with markers for IFN-γ, TNF, perforin, granzyme A, granzyme B and CD107a. Gating strategies are shown in **Supplemental Figure 4**. For this analysis we examined: granzyme A, granzyme B and perforin levels in untreated samples; CD107a expression using K562 co-cultured cells; and IFN-γ and TNF production in IL-12/IL-18 treated cells (n=6).

To undertake the analysis, we looked at patterns of these different NK cell markers before and after ART compared to HIV-uninfected participants (n=8). **Figures 1D, E** shows only those markers where there was a statistical difference for at least one of the comparisons; all other analyses are presented in the Supplementary Materials. We found three general patterns. The first pattern included markers that were impacted by HIV infection (i.e. significantly different between PWH and HIV-uninfected individuals off ART) but then normalised over 2 years of ART (**Figure 1D**). These markers included at baseline: a higher fraction of Eomes-/T-bet- CD56^dim^ NK cells, lower NKG2D levels on CD56^dim^ NK cells, higher CD38 levels on CD56^bright^ NK cells, higher NKG2A expression on CD56^bright^ NK cells, increased granzyme A levels on untreated CD56^bright^ NK cells and reduced IFN-γ expression in cytokine stimulated CD56^neg^ NK cells.

The second pattern included markers which were different in HIV infected individuals at baseline compared with HIV uninfected participants, and maintained that difference after two years of ART (**Figure 1E**). In HIV-infected individuals, these markers included a lower fraction of Eomes-/T-bet++ CD56^dim^ NK cells, higher expression of NKG2C on CD56^dim^ cells, reduced IFN-γ expression in cytokine stimulated CD56^dim^ NK cells, a lower fraction of Eomes-/T-bet+ CD56^bright^ NK cells, higher expression of Tim-3 on CD56^bright^ cells, and a higher percentage of Eomes-T-bet- CD56^neg^ cells. There was also a trend for a baseline difference in NKG2A expression on CD56^neg^ cells (p=0.061), which did not reverse after 2 years of ART (**Figure 1E**).

The third pattern comprised NK cell markers with no statistical difference between HIV infected and uninfected individuals at baseline or 2 years after ART (**Supplemental Figures 5 and 6**).

There was no statistical difference in any marker in PWH between samples at baseline and 12 weeks after ART initiation suggesting any ART mediated recovery of NK cell phenotype and function was not immediate (**Figure 1**). We did see a trend toward lower CD38 levels in CD56^bright^ NK cells after 12 weeks of ART (**Figure 1D** p=0.055) suggesting activation levels were the first to decrease after ART initiation.

### NK cell phenotype and time to viral rebound

Having explored the impact of ART on NK phenotype and function, we turned to the second part of the study and a different cohort to determine if those NK markers identified in the first part were associated with correlates of HIV reservoir size and dynamics of viral rebound following ART cessation. We studied 44 participants from the SPARTAC trial [22] who received 48 weeks of ART and then stopped therapy. Their summarised clinical characteristics are shown in **Table 1**. Median baseline viral load (IQR) was 45,643 copies/mL (15,414-15,125), baseline CD4 T cell count was 644 cells/μL (456-771.5), median time to viral rebound was 6 weeks (1-40) and median total HIV DNA copies/million CD4 T cells at treatment interruption was 1749 (1087-2762). Median estimated time from seroconversion to ART initiation was 90.5 days (66-110.3)

**Figure 2:**
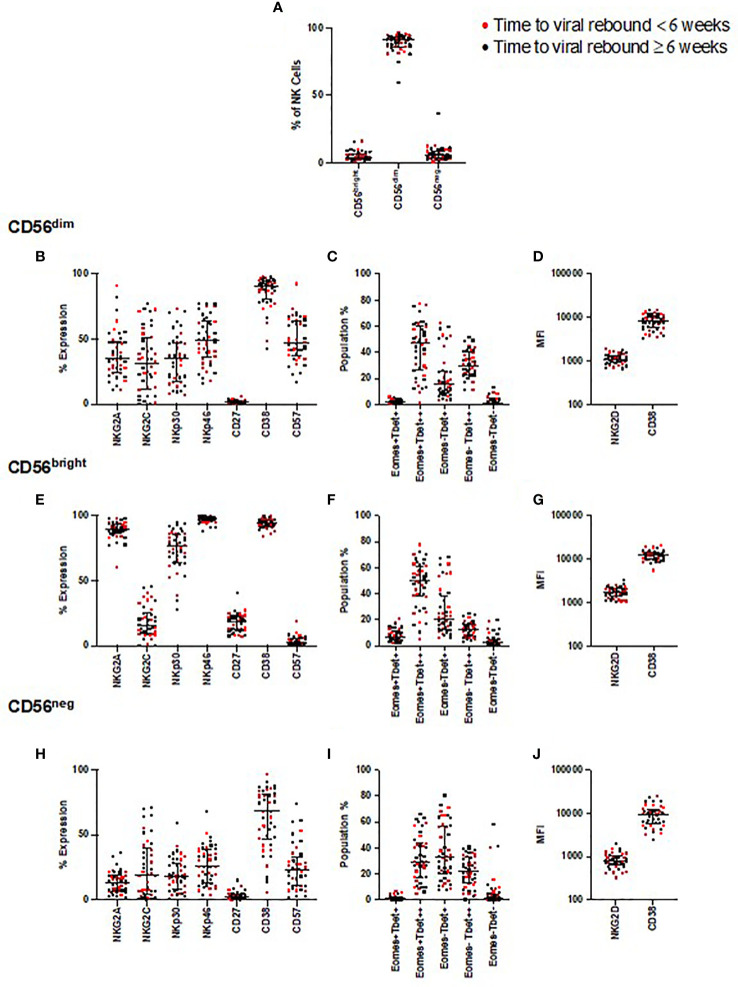
NK cell phenotyping and time to viral rebound. PBMC from SPARTAC patients (n = 44) were stained and divided into CD56^dim^, CD56^bright^, and CD56^neg^ NK cells **(A)**. Red circles indicate individuals with time to viral rebound < 6 weeks and black circles indicate individuals with time to viral rebound ≥ 6 weeks. Expression as a percent or median fluorescent intensity is shown for CD56^dim^ NK cells **(B–D)**, CD56^bright^ **(E–G)** and CD56^neg^ NK cells **(H–J)**. For all graphs, median values with interquartile range are shown. A blue box represents those that were different at baseline but corrected over 2 years of ART, a red box represents those that were different at baseline but which did not correct after two years of ART.

We first examined whether the relative frequencies of CD56^bright^, CD56^dim^, and CD56^neg^ NK cells correlated with time to viral rebound or were enriched in individuals with faster or slower times to viral rebound. For this latter analysis the dataset was divided into two groups based on the median time to viral rebound, < or ≥ 6 weeks. We found no correlation with time to viral rebound or any difference for any of these NK cell populations between the two groups (**Figure 2A** and **Supplemental Table 1**).

We examined expression of the same NK cell phenotypic markers used in the longitudinal study, and looked for associations with time to viral rebound. We found no correlations with any of these phenotypic markers and time to rebound (**Figure 2**) nor did we find enrichment of these markers in those with < or ≥ 6 weeks to viral rebound (**Supplemental Table 1**).

As the participants demonstrated a range of times to viral rebound, we next focused on those with the longest period of PTC, in case they represented an extreme phenotype. We identified those participants whose time to viral rebound was >1 year after TI (n=8), a cut-off similar to other PTC studies (32, 33). We found no distinct separation of individuals with viral rebound after one year compared to the rest of the cohort for any phenotypic marker we examined (**Supplemental Figure 7**). Instead, we found these individuals were distributed among the rest of the cohort, suggesting no single NK cell marker defined PTC in these participants.

### NK cell function and time to viral rebound

**Figure 3:**
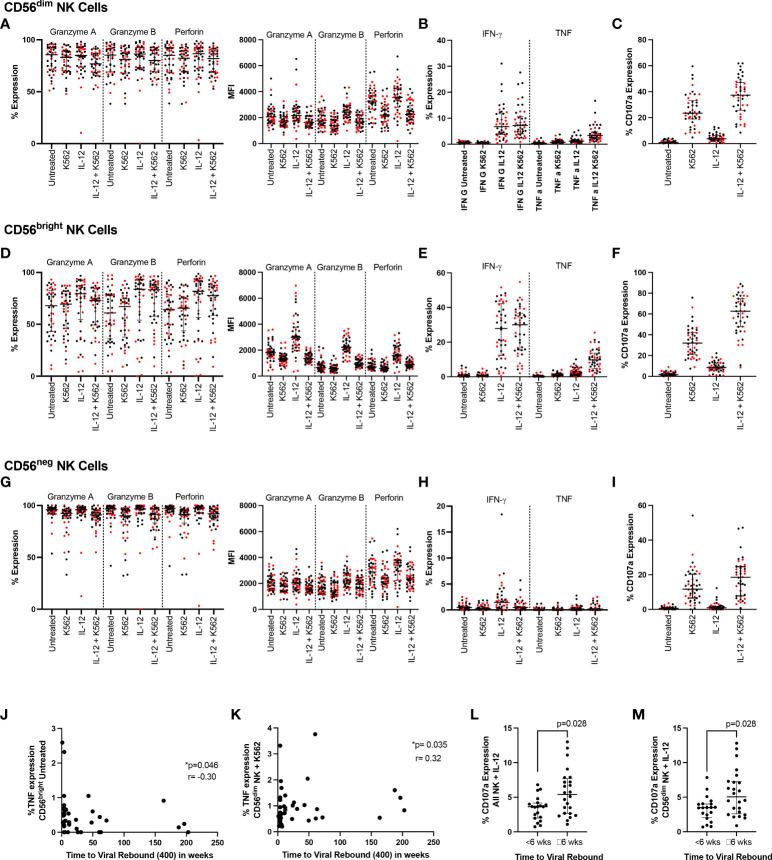
NK cell function and time to viral rebound. PBMC from SPARTAC participants (n = 44) were either cultured overnight in media or stimulated with 10ng/mL IL-12 and 100ng/mL IL-18. Cells were then cultured alone or with K562 cells at a 5:1 E:T ratio for 5 hours in the presence of CD107a. Cells were then stained intracellularly for granzyme **(A)**, granzyme **(B)**, perforin, IFN-γ and TNF. Red circles indicate individuals with time to viral rebound < 6 weeks and black circles indicate individuals with time to viral rebound ≥ 6 weeks. Expression as a percent or median fluorescent intensity is shown for CD56^dim^ NK **(A–C)**, CD56^bright^ NK **(D–F)** and CD56^neg^ NK cells **(G–I)**. In **(J)** and **(K)**, time to viral rebound was correlated with TNF expression in untreated CD56^bright^ NK cells and TNF expression in untreated CD56^dim^ NK cells co-cultured with K562 cells, respectively. A Spearman test was used and p and r values are shown. **(L)** and **(M)** show comparisons between individuals with time to viral rebound < 6 weeks compared to ≥ 6 weeks for CD107a expression in all NK cells **(L)** and CD56^dim^ NK cells **(M)** stimulated with IL-12 and IL-18. For all graphs, median values with interquartile range are shown.

In addition to the K562 and IL-12/18 stimulation used in the longitudinal experiments, we also combined the models to examine the effect of activating NK cells with cytokines before co-culturing them with K562 cells (**Figures 3A–I**). We examined all markers in all conditions and compared participants who rebounded either within 6 weeks (red markers in **Figures 3A–I**) or ≥6 weeks (black markers in **Figures 3A–I**). Examining functional responses in CD56dim (**Figures 3A–C**), CD56bright (**Figures 3D–F**) and CD56neg (**Figures 3G–I**) NK cells, we found no difference according to this categorical time to rebound.

We then examined whether any functional markers were associated with time to viral rebound as a continuous function. We found baseline TNF expression in untreated CD56^bright^ NK cells negatively correlated with time to viral rebound (**Figure 3J** p=0.046, r=-0.302) while TNF levels in CD56^dim^ NK cells co-cultured with K562 cells positively correlated with time to viral rebound (**Figure 3K** p=0.035, r=0.319). We also saw higher levels of CD107a in bulk NK cells and CD56^dim^ NK cells treated with IL-12 and IL-18 in individuals who rebounded ≥ 6 weeks (**Figures 3L, M**, p=0.028 for both). However, we did not see a difference when cells were co-cultured with K562 cells, suggesting this was a potentially small difference that was overcome with a stronger degranulation stimulus (**Supplementary Table 1**).

We again examined whether individuals who rebounded after a year off ART clustered separately. Similar to our phenotypic markers, we found those who rebounded after a year were distributed among the rest of the cohort suggesting no clear PTC phenotype based on NK cell function (**Supplemental Figure 8**).

### NK cell phenotype and HIV DNA levels

**Figure 4:**
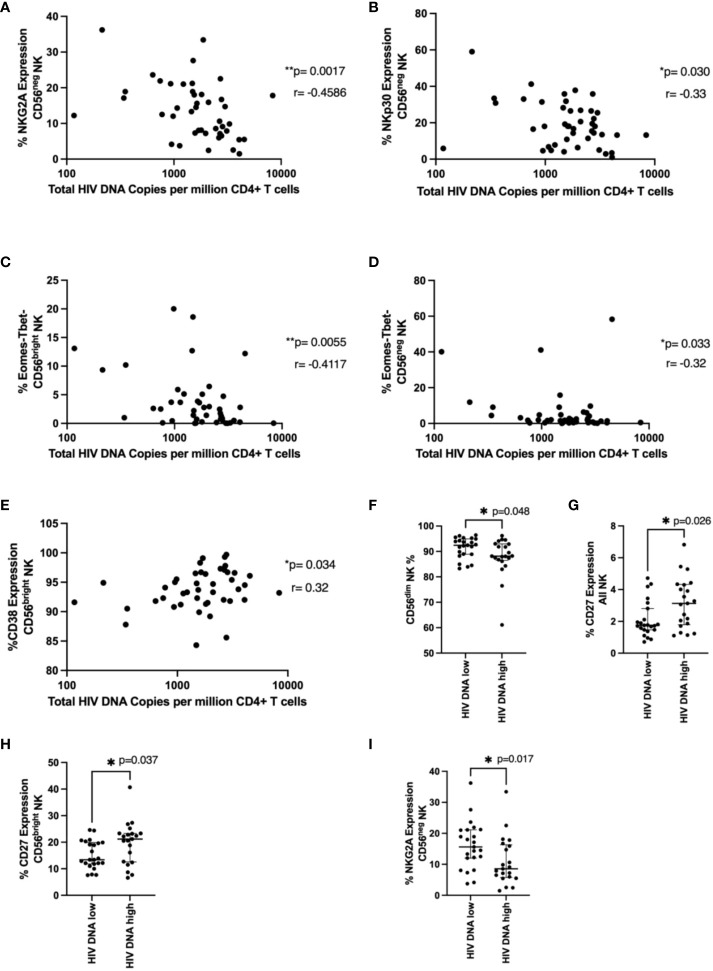
NK cell phenotype and HIV DNA levels. In **(A–E)**, total HIV DNA at 48 weeks (log scale) was correlated with the indicated NK cell marker in the indicated NK cell population using a Spearman correlation. p and r values are shown. In **(F–I)**, a Mann-Whitney test was performed between individuals with < or ≥ median HIV DNA levels (1749 HIV copies/million CD4 T cells) for the indicated marker and NK cell population. p values are shown. * represents p < 0.05, ** represents p < 0.01 (n = 44).

Next, we examined NK cell phenotypic markers and their relationship with total HIV DNA levels measured in bulk CD4 T cells at the time of ART interruption. NKG2A and NKp30 expression in the CD56^neg^ population negatively correlated with HIV DNA (**Figures 4A, B**; p=0.00174, r=-0.459 and p=0.03, r= -0.327, respectively). The Eomes-T-bet- population in both CD56^bright^ and CD56^neg^ NK cells also negatively correlated with HIV DNA (**Figures 4C, D** p=0.0055 r=-0.41, p=0.033 r=-0.32 respectively). Expression of the activation marker CD38 in the CD56^bright^ population positively associated with HIV DNA (**Figure 4E** p=0.034, r=0.32).

We next wanted to see if specific NK cell phenotypic markers were enriched in those with higher or lower levels of HIV DNA. Similar to our time to viral rebound studies we divided our cohort into those with < or ≥ median HIV DNA levels of 1749 HIV copies/million CD4 T cells. We found significantly higher fractions of CD56^dim^ NK cells in individuals with lower HIV DNA levels (**Figure 4F**, p=0.048) suggesting cytotoxic NK cells may be important for limiting HIV DNA levels. We also found levels of CD27 positive NK cells and CD56^bright^ NK cells were significantly higher in individuals with higher levels of HIV DNA (**Figures 4G, H**, p=0.026 and p=0.037 respectively). Previous studies have shown upregulation of CD27 during HIV infection, particularly during chronic HIV infection (20). Finally, we also saw significantly lower levels of NKG2A in CD56^neg^ NK cells in patients with higher HIV DNA levels (**Figure 4I**, p=0.017), supporting the findings from the correlation analysis.

### NK cell function and HIV DNA levels

**Figure 5:**
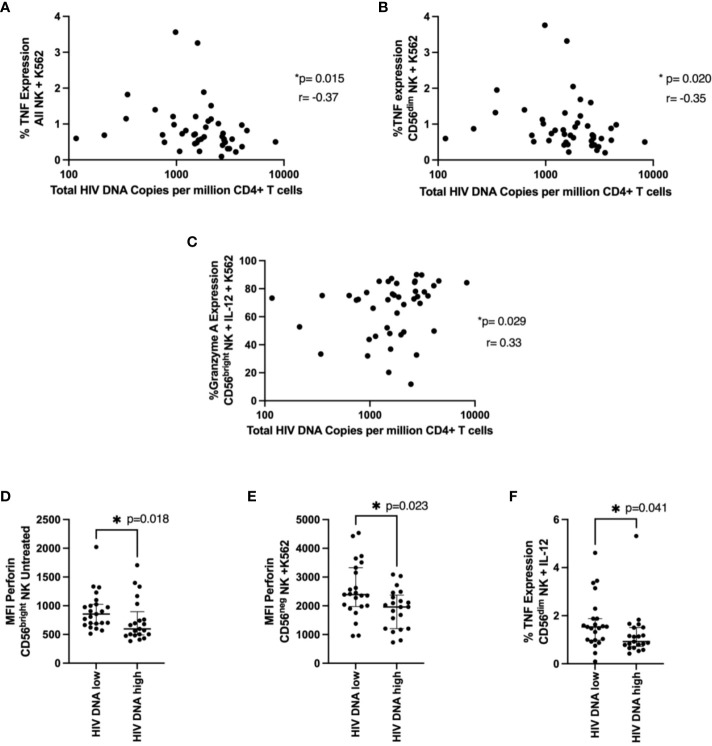
NK cell function and total HIV DNA levels. Cells were treated and stained as in **Figure 4**. In **(A, B),** a correlation between TNF expression in NK cells **(A)** or in CD56^dim^ NK cells **(B)** co-cultured with K562 cells and total HIV DNA at 48 weeks post ART is shown. A Spearman test was used and p and r values are shown. In **(C–F)** a Mann-Whitney test was performed between individuals with < or ≥ median HIV DNA levels for the MFI of perforin in untreated CD56^bright^ NK cells **(C)**, the MFI of perforin in CD56^neg^ NK cells co-cultured with K562 cells **(D)** and the percent TNF expression of CD56^dim^ NK cells treated with IL-12 and IL-18 **(E)**. *P < 0.05

We examined the relationship between NK functional markers and HIV DNA levels. We found expression of TNF after K562 co-culture in NK cells and CD56^dim^ NK cells negatively correlated with HIV DNA levels (**Figures 5A, B**, p=0.015 r=-0.37, p=0.02 r=-0.35 respectively), consistent with their positive association with time to viral rebound. Granzyme A expression also positively correlated with HIV DNA levels in cytokine treated cells co-cultured with K562 (**Figure 5C**, p=0.029, r=0.33). We also found significantly higher levels of perforin (MFI) in individuals with lower HIV DNA levels in both untreated CD56^bright^ NK cells (**Figure 5D**, p=0.018) and CD56^neg^ NK cells co-cultured with K562 cells (**Figure 5E**, p=0.023), again suggesting a role for cytotoxic NK cells in limiting HIV DNA. Finally, we saw significantly higher TNF expression in cytokine stimulated CD56^dim^ NK cells in individuals with lower HIV DNA levels (**Figure 5F**, p=0.041).

### Principal component analysis (PCA)

**Figure 6:**
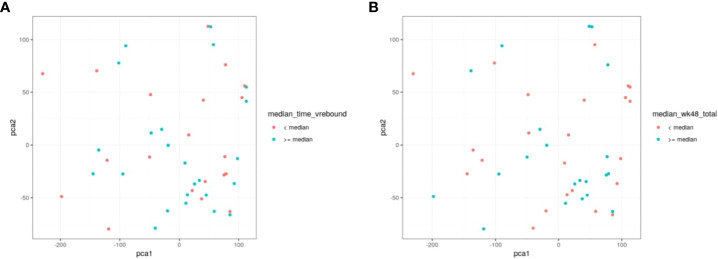
PCA does not discriminate between individuals based on time to viral rebound or total HIV DNA. NK cell markers and viral measurements were combined and analysed using PCA. In **(A)** red circles represent individuals with time to viral rebound < 6 weeks and blue circles indicate individuals with time to viral rebound ≥ 6 weeks. In **(B)** red circles represent individuals with HIV DNA levels < median and blue circles indicate individuals with ≥median HIV DNA levels.

To further evaluate our cohort with regard to time to viral rebound and HIV DNA, we turned to PCA analysis. We found no statistical separation between individuals with time to viral rebound < or ≥ 6 weeks (**Figure 6A**) or when examining groups divided around median HIV DNA levels (**Figure 6B**).

### Cytomegalovirus (CMV) infection

As CMV co-infection is highly prevalent in HIV-1 cohorts and has been shown to greatly affect NK cell phenotype (34), we wanted to examine the potential impact of CMV on our findings. First, we included NKG2C in our panel to help evaluate the effects of CMV as NKG2C expression on NK cells is known to be upregulated by CMV infection (34). NKG2C neither correlated with HIV DNA nor time to viral rebound (**Supplemental Table 1**). Additionally, we also measured CMV titres in our SPARTAC cohort and found that patients with higher CMV titres had no statistically different HIV DNA levels or time to viral rebound than those with lower titres (**Supplemental Figure 9**).

## Discussion

Natural Killer cells are potential key players in the road to achieving HIV cure or sustained virological remission off ART. Their ability to kill HIV infected cells (9, 11), links to non-pathogenic animal models of SIV (12), correlations with vaccine protection (25, 26), and possible roles in controlling HIV DNA levels in HIV controllers and intervention studies (13, 14) all provide evidence supporting the important role NK cells may play in controlling HIV. More recent studies have also suggested NK cells may affect the induction of broadly neutralising antibody responses to HIV (10) and may have immune memory (27) which could potentially play a role in controlling viral rebound and post treatment control.

PTC provides a potential goal for HIV cure strategies, allowing individuals to remain safely off antiretroviral therapy for long periods of time. Understanding the biological factors affecting time to viral rebound would provide crucial information for both treatment interruption studies as well as therapeutic targets. However, factors dictating viral rebound or predicting who can be safely taken off therapy have yet to be elucidated (21).

As PTC is more likely in those commencing ART during primary compared with chronic HIV infection (3, 21), we examined how NK cell properties were affected over time following early ART. The majority of other studies examining the effect of ART on NK cells are focused on chronic infection, are not longitudinal or were after long periods of ART (18–20, 28). As we have previously reported evidence for PTC after 48 weeks of therapy (15), our data suggests that extremely long ART periods may not be required for PTC. Thus, we took a small group of individuals who received ART during primary HIV infection and intensively studied the phenotype and function of their NK cells longitudinally at baseline (ART naïve), 3 months, 1 year, and 2 years post ART.

We first examined NK cell subsets based on CD56 expression with gating strategies dividing cells into CD56^bright^, CD56^dim^, and CD56^neg^, similar to Costanzo et al (29). CD56^bright^ cells are typically less cytotoxic and have higher proliferative potential. CD56^dim^ cells are more cytotoxic (30), while CD56^neg^ cells are dysfunctional cells that increase during HIV infection (31, 35). Similar to some other studies, we found no significant differences in the fraction of CD56^bright^ cells but lower levels of CD56^dim^ cells in untreated HIV infection (36). We also found a trend towards higher fractions of CD56^neg^ NK cells at baseline in HIV infected individuals which is well reported (17). All of these differences in NK cell subset distribution disappeared after 2 years of ART.

We next examined phenotypic and functional NK cell markers in these different populations longitudinally after ART initiation using two stimulation models and found several markers that were restored by ART and several that did not return to pre-ART levels. Markers were chosen to represent a broad coverage of activation and exhaustion as well as activating and inhibiting receptors (as well as NKG2C as a surrogate for CMV serostatus). Any flow cytometry methodology is restricted by the number of markers that are available and newer technologies such as CyTof would permit a much more detailed analysis. Patterns of T-bet and Eomes were significantly changed by HIV infection and were still significantly different from uninfected controls despite 2 years of ART (**Figure 1**). This was likely due to the formation of a Eomes-/T-bet- population caused by transcription factor downregulation by HIV (37). Importantly these transcription factors are important for NK function and cytolytic activity (38). NK cell activation as measured by CD38 was similar to those in uninfected individuals post therapy and was the only marker to trend towards restoration after only 3 months of ART. Other markers with this expression pattern included a combination of activating (NKG2D levels on CD56^dim^ NK cells) and inhibitory receptors (NKG2A expression on CD56^bright^ NK cells) and functional markers (granzyme A levels in CD56^bright^ NK cells and reduced IFN-γ expression in cytokine stimulated CD56^neg^ NK cells).

Other markers that were still different in PWH and uninfected individuals after 2 years of therapy included higher expression of NKG2C on CD56^dim^ cells, likely due to CMV infection in HIV-infected individuals which is known to upregulate NKG2C (34). We also observed higher expression of Tim-3 on CD56^bright^ cells in ART treated PWH individuals compared to uninfected controls (**Figure 1K**). Tim-3 has been previously shown to be a marker of NK exhaustion (37, 39, 40) and may indicate a potentially irreversibly exhausted population of NK cells.

As our longitudinal analysis used separate uninfected controls rather than the pre-HIV samples, differences between the cohorts such as CMV serostatus and sex may contribute to the differences in NK cell marker expression. However, by comparing multiple timepoints during HIV infection with uninfected samples we were able to minimise the contribution of these confounders. By comparing pre-ART and post-ART samples with uninfected controls, markers that changed after ART exposure were likely not caused by cohort differences; if cohort characteristics were responsible, differences in NK cell marker expression would be present both pre- and post- therapy. That there are clear differences in the reversibility of some marker changes over time on ART remains of interest, and also that this may vary between individuals. Whether this is determined by differing levels of very low grade viraemia, differences in reservoir size and depth of latency (and potential expression of antigen on latently infected cells), levels of Nef-induced class I down-regulation, or degrees of chromatin accessibility remains to be seen. However, the fact that post-treatment control is rare might suggest that it is worth focusing on those characteristics that revert in a few individuals, although in this study we were unable to identify a link. For this, much larger studies would be needed.

After observing these differences in NK cell phenotype and function during primary HIV infection, we turned to the SPARTAC treatment interruption trial to determine if NK cells might play a role in controlling HIV reservoir size or delaying time to viral rebound. SPARTAC was ideal as it was a large randomised controlled trial where we had previously examined the role of T cells and viral factors on time to viral rebound (24, 41). We studied both NK cell phenotypic and functional markers and compared these markers with time to viral rebound and HIV DNA levels.

We found no NK cell phenotypic marker correlated with time to viral rebound and only weak functional correlations with TNF expression that were not statistically significant after correcting for multiple comparisons. Similarly, we found only CD107a expression was differentially expressed between two populations divided around median time to viral rebound. These data suggest none of the markers we examined effectively predicted time to viral rebound. Nor were we able to separate our cohort based on time to viral rebound using PCA analysis (**Figure 6**).

We also identified NK cell markers that related to levels of HIV DNA at the time of treatment interruption suggesting the importance of functional NK cells and the potential consequences of dysregulated, dysfunctional NK cells. For example, NKG2A expression in CD56^neg^ NK cells univariably negatively correlated with HIV DNA levels (**Figure 3A**) and this corresponded with an enrichment of this marker in patients with HIV DNA levels below the median (**Figure 3I**). As the downregulation of NKG2A on CD56^neg^ NK cells has been previously associated with NK dysfunction (42), our data is consistent with a role for functional NK cells limiting HIV DNA levels. We also found an enrichment of the marker CD27 in all NK and CD56^bright^ NK cells in those with higher HIV DNA levels (**Figures 3G, H**). This population has been associated with higher IFN-γ production in viral infection, particularly HCV (43) and has been shown to be dysregulated in HIV (20). Our data also suggests a beneficial role of cytotoxic NK cells as we observed enrichment of the more cytotoxic CD56^dim^ NK cell population in individuals with lower HIV DNA (**Figure 3F**) and that increased perforin levels in CD56^bright^ and CD56^neg^ NK cells were found in individuals with lower HIV DNA (**Figures 5D, E**).

These identified markers did not share a similar pattern with regards to the effects of ART: some were not different off and on ART, some had similar expression compared to uninfected controls after 2 years of ART and others were still significantly different to uninfected individuals despite 2 years of ART, suggesting markers relating to viral rebound and control may not be those most affected by ART. Our data also suggest that NK cell factors involved in time to viral rebound may not be the same as those affecting HIV DNA levels as we did not see much overlap between factors affecting HIV DNA levels compared with those affecting time to viral rebound. This could be due to HIV protein production by replication incompetent virus in the absence of viral rebound (44). We also examined those individuals who rebounded after a year off ART in case they presented a unique phenotype. However, these individuals did not cluster for any examined marker suggesting they did not present a unique NK cell profile based on the markers studied.

There are limitations to this study. First, due to limited sample availability, we did not examine HIV specific responses, instead relying on cytokine stimulation and K562 co-culture to evaluate HIV function. Additionally, our use of flow cytometry rather than a higher output technique such as CyTOF or transcriptomics meant our study was not designed for identifying specific subsets of NK cell subsets based on multiple markers. Recent studies have suggested these types of populations may be important in understanding the role of NK cells in HIV infection (29, 45). Equally, due to sample limitations we were unable to perform functional assays for ADCC which would have added interest in the context of cure strategies incorporating broadly neutralising antibodies. As we had previously studied immune response in SPARTAC, we did not have sufficient baseline samples for analysis and so relied on samples collected 12 weeks post ART initiation for analysis. However, our longitudinal HEATHER data indicated no statistical differences between these timepoints for the markers we examined. It is also possible that different NK cell markers at the time of treatment interruption may affect time to viral rebound. Furthermore, we used total HIV DNA measured in CD4 T cells as a surrogate marker of reservoir size. It is now well established that this will over-estimate the size of the replication competent reservoir due to the incorporation of defective genomes (46). Although we acknowledge that total HIV DNA is not an accurate measure of the reservoir, it still has potential value as a biomarker for viral burden or antigenic challenge and we previously found total HIV DNA related to time to viral rebound in previous SPARTAC analysis (24). Although we were able to include an HIV-ve control group, we were not able to ensure matching for age and gender; as such there may be unrecognised confounders in the analysis, although this could only really be assessed by undertaking detailed immunological analyses in the context of a randomised controlled trial. Finally, the SPARTAC cohort was a mix of individuals from different regions with different HIV clades and gender. This made teasing apart the effects of these factors difficult as we have previously reported (47). However, our focus was on identifying differences in NK cell properties that could affect HIV across the entire cohort rather than comparing clades or differences between different countries.

Overall, our work indicates that while early ART started in PHI can restore a number of changes to NK cell phenotype and function imposed by HIV infection, this is not complete. While there is evidence that NK cells may play a role in delaying viral rebound and limiting HIV DNA levels, we could not identify individual markers that predicted time to viral rebound or that could separate our cohort around median time to viral rebound or HIV DNA level. These data are important as with large numbers of clinical trials and translational studies exploring HIV cure strategies, identifying key immune effector populations is going to be crucial to success. The fact that we were unable to identify an NK cell phenotype that could be a potential driver of HIV remission does not mean that one does not exist, but does suggest that well-defined large studies and collaborative efforts may be needed to identify those NK cells that can prevent viral rebound on stopping ART.

## Data availability statement

The original contributions presented in the study are included in the article/**Supplementary Material**. Further inquiries can be directed to the corresponding author.

## Ethics statement

The studies involving human participants were reviewed and approved by West Midlands—South Birmingham Research Ethics Committee (reference 14/WM/1104). The patients/participants provided their written informed consent to participate in this study.

## Author contributions

JFr, MP, SF, and JFo were involved in conceptualization, data curation, funding acquisition, supervision, methodology, writing and reviewing the manuscript. MP, AO, JH, NR, JM, NO, JT, MJ, AW, JL, KK, RH, PZ, GM, HB, NN, and DP were involved with sample methodology and preparation, assay performance, data curation, data analysis and writing and reviewing the manuscript. SF managed study recruitment at Imperial; AW, JL, and JFo managed study recruitment at Guy’s. All authors critically reviewed and approved the final version.

## Funding

This work was funded by the MRC through grants to JFr and MP (MR/L006588/1 and MR/P011233/1).

## Conflict of interest

Author JH was employed by Etcembly Ltd, Harwell Campus, United Kingdom.

The remaining authors declare that the research was conducted in the absence of any commercial or financial relationships that could be construed as a potential conflict of interest.

## Publisher’s note

All claims expressed in this article are solely those of the authors and do not necessarily represent those of their affiliated organizations, or those of the publisher, the editors and the reviewers. Any product that may be evaluated in this article, or claim that may be made by its manufacturer, is not guaranteed or endorsed by the publisher.
