# Supplementary material for: Impact of antiretroviral therapy in primary HIV infection on natural killer cell function and the association with viral rebound and HIV DNA following treatment interruption
Source: Front Immunol. 2022 Aug 30;13:878743. doi: 10.3389/fimmu.2022.878743 (PMC9468877; doi:10.3389/fimmu.2022.878743)
Supplement: Supplementary file 1 [file DataSheet_1.docx]

**Supplementary Methods.** The SPARTAC trial was approved by the following authorities:

the Medicines and Healthcare products Regulatory Agency (UK), the Ministry of

Health (Brazil), the Irish Medicines Board (Ireland), the Medicines Control Council

(South Africa) and the Uganda National Council for Science and Technology

(Uganda). It was also approved by the following ethics committees in the

participating countries: the Central London Research Ethics Committee (UK),

Hospital Universitário Clementino Fraga Filho Ethics in Research Committee

(Brazil), the Clinical Research and Ethics Committee of Hospital Clinic in the

province of Barcelona (Spain), the Adelaide and Meath Hospital Research Ethics

Committee (Ireland), the University of Witwatersrand Human Research Ethics

Committee, the University of Kwazulu-Natal Research Ethics Committee and the

University of Cape Town Research Ethics Committee (South Africa), Uganda Virus

Research Institute Science and Ethics Committee (Uganda), the Prince Charles

Hospital Human Research Ethics Committee and St Vincent’s Hospital Human

Research Ethics Committee (Australia) and the National Institute for Infectious

Diseases Lazzaro Spallanzani, Institute Hospital and the Medical Research Ethics

Committee, and the ethical committee of the Central Foundation of San Raffaele,

MonteTabor (Italy).


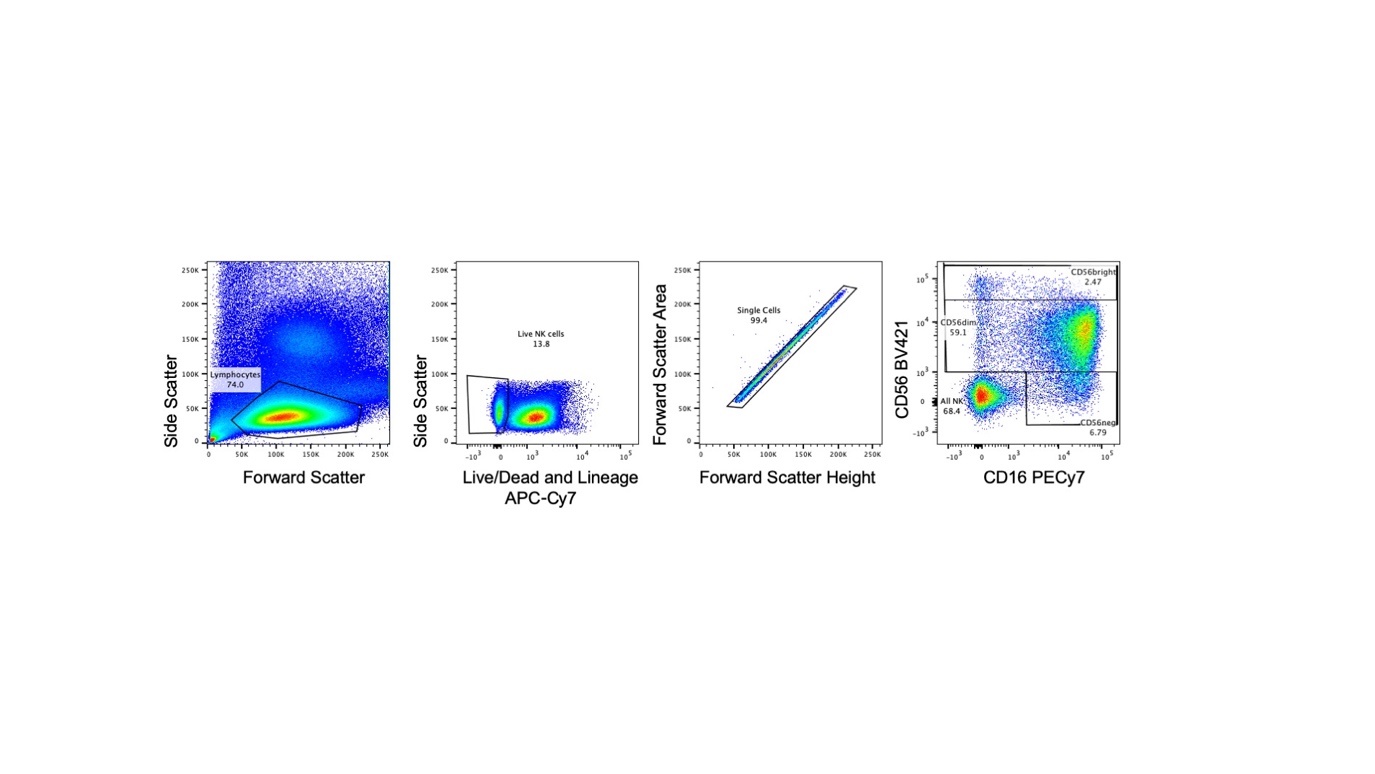


**Supplemental Figure 1. NK subset gating strategy.** Lymphocytes were gates on forward and side scatter. A dump channel including a live/dead stain, CD3, CD14, and CD19 were used to exclude dead cells, T cells, B cells, and monocytes. Singlets were then gated based on forward scatter area and height. NK subsets were then gated based on CD56 and CD16 expression.


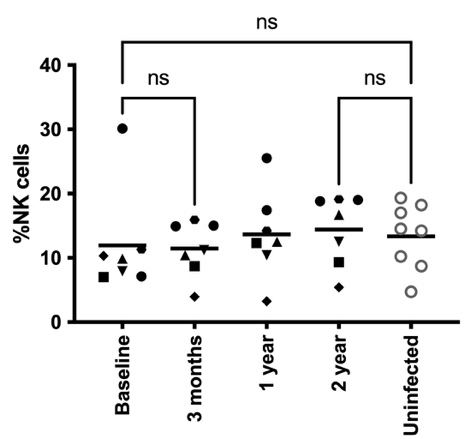


**Supplemental Figure 2. NK cell frequency.** The frequency of live NK cells was calculated as a fraction of lymphocytes using flow cytometry and compared over time. A Kruskal-Wallis test with Dunn’s multiple comparison test was performed between baseline and 3 months ART, baseline and uninfected samples, and 2 year and uninfected samples.

**
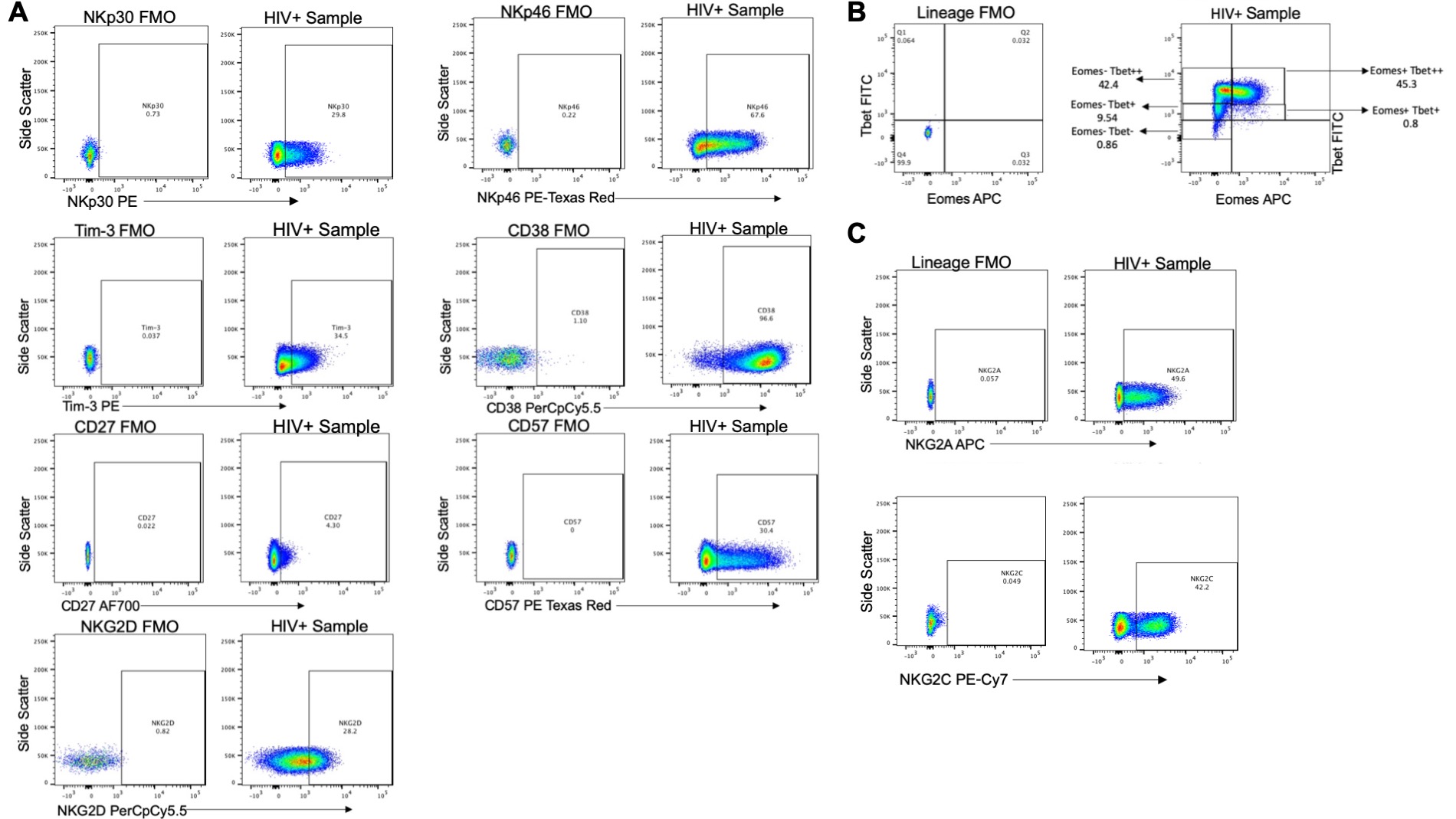
**

**Supplemental Figure 3. NK phenotype staining gating strategy.** NK cells gated as above were labelled with various phenotypic markers. **A** shows those markers where the gate was set using marker specific ‘Fluoresence Minus One’ (FMO) gates on the left and fully stained HIV+ samples are shown are the right. **B** shows the gating strategy for the transcription factor gating strategy for Tbet and Eomes and the 5 resulting populations: Eomes+Tbet++, Eomes+ Tbet+, Eomes- Tbet++, Eomes- Tbet+ and Eomes-Tbet-. **C** shows those markers who gating was based on a generic lineage FMO including CD56 and CD16.

**
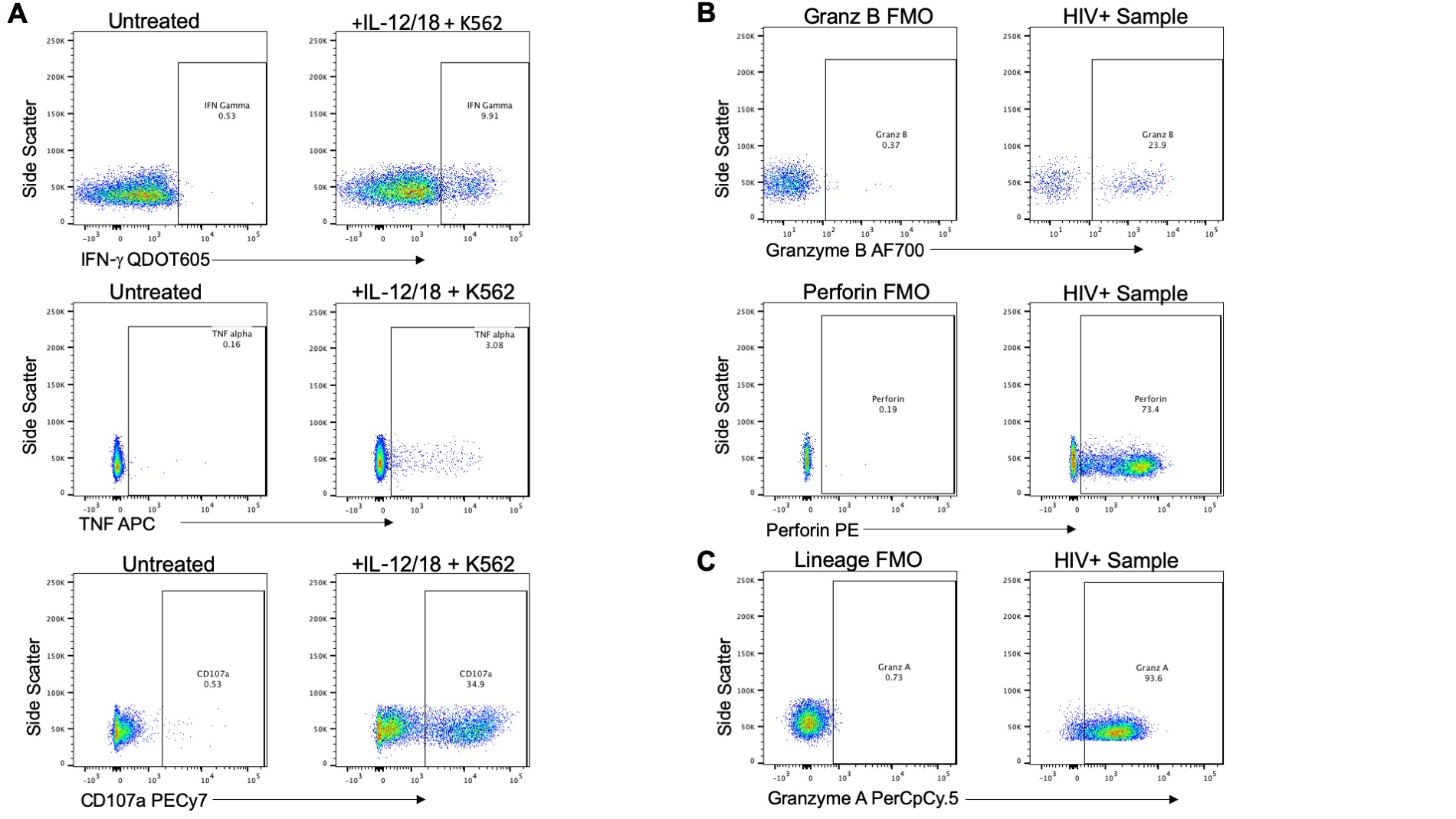
**

**Supplemental Figure 4. Functional NK Gating Strategy.** Gating strategy for functional markers is shown. IFN-γ , TNF and CD107a were gated using samples cultured in media alone without K562 cells (untreated) with samples stimulated with IL-12/18 and co-cultured with K562 cells shown for comparison **(A)**. Granzyme B and perforin were gated based on marker specific FMOs with untreated HIV+ samples serving as a comparison **(B).** In C the gating strategy for granzyme A is shown using a lineage FMO.

**Supplemental Figure 5. NK phenotypic markers longitudinally on ART.** PBMC from HEATHER samples were taken at baselined (off ART), 3 months, 1 year and 2 years post ART and compared to HIV negative individuals. Experiments were performed as in Figure 1. Phenotypic markers as percent expression or median fluorescence intensity (MFI) are shown for HEATHER samples (n=7) and HIV- samples (n=8) for CD56^dim^ (**A**), CD56^bright^ (**B**), and CD56^neg^ NK cells (**C**). Only samples without statistical differences are shown. A Kruskal-Wallis test with Dunn’s multiple comparison test was performed between baseline and 3 months ART, baseline and uninfected samples, and 2 year and uninfected samples.

**Supplemental Figure 6. NK cell function longitudinally on ART.** PBMC from HEATHER samples were taken at baselined (off ART), 3 months, 1 year and 2 years post ART and compared to HIV negative individuals. Functional experiments were performed as in Figure 1. Phenotypic markers as percent expression or median fluorescence intensity (MFI) are shown for HEATHER samples (n=7) and HIV- samples (n=8) for CD56^dim^ (**A**), CD56^bright^ (**B**), and CD56^neg^ NK cells (**C**). Only samples without statistical differences are shown. A Kruskal-Wallis test with Dunn’s multiple comparison test was performed between baseline and 3 months ART, baseline and uninfected samples, and 2 year and uninfected samples.

**Supplemental Figure 7. NK phenotype in patients with viral rebound >1 year.** The data shown are identical to those in Figure 1 with black x represent individuals who rebound in less than 52 weeks and red circles represent those who rebounded at or after 52 weeks post ART interruption.

**Supplemental Figure 8. NK function in patients with viral rebound > 1 year.** The data shown are identical to those in Figure 3 with black x represent individuals who rebound in less than 52 weeks and red circles represent those who rebounded at or after 52 weeks post ART interruption.


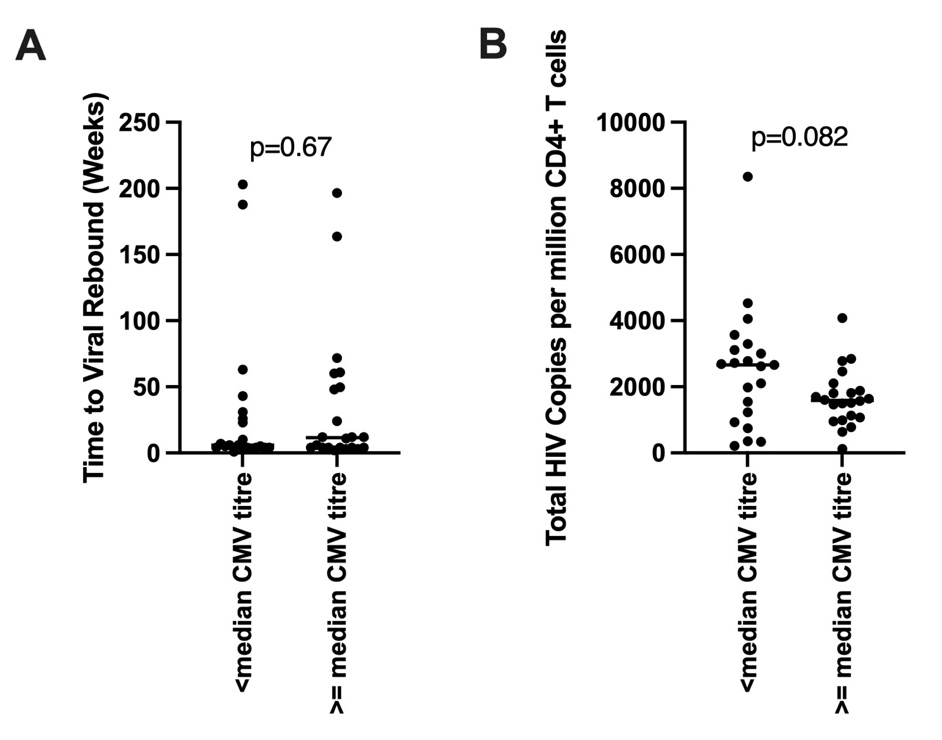


**Supplemental Figure 9. CMV titres and time to viral rebound and total HIV DNA levels.** Time to viral rebound (A) and total HIV (B) were compared between patients with < median CMV titres (168.6 AU/mL) and those with ≥ median CMV titres. Values were compared using Mann-Whitney test. p values are shown.

**Supplemental Figure 10.** Detailed statistics from Figure 1 in the manuscript showing comparisons (uncorrected) across all time-points for those markers showing changes over time on ART.
